# Linking fishes to multiple metrics of coral reef structural complexity using three-dimensional technology

**DOI:** 10.1038/s41598-017-14272-5

**Published:** 2017-10-25

**Authors:** M. González-Rivero, A. R. Harborne, A. Herrera-Reveles, Y.-M. Bozec, A. Rogers, A. Friedman, A. Ganase, O. Hoegh-Guldberg

**Affiliations:** 1 The Global Change Institute, The University of Queensland, St Lucia, Queensland, 4072 Australia; 2 Australian Research Council Centre of Excellence for Coral Reef Studies, The University of Queensland, St Lucia, Queensland, 4072 Australia; 3 Department of Biological Sciences, Florida International University, North Miami, Florida, 33181 USA; 4 Instituto de Zoología y Ecología Tropical, Universidad Central de Venezuela. Caracas, Distrito Capital, 1051 Venezuela; 5 School of Biological Sciences, The University of Queensland, St Lucia, Queensland, 4072 Australia; 6 Greybits Engineering, Sydney, New South Wales, 2029 Australia; 7 The Australian Centre for Field Robotics, University of Sydney, New South Wales, 2006 Australia; 8 Present Address: Australian Institute of Marine Science, PMB 3, Townsville MC, Queensland, 4810 Australia

## Abstract

Structural complexity strongly influences biodiversity and ecosystem productivity. On coral reefs, structural complexity is typically measured using a single and small-scale metric (‘rugosity’) that represents multiple spatial attributes differentially exploited by species, thus limiting a complete understanding of how fish associate with reef structure. We used a novel approach to compare relationships between fishes and previously unavailable components of reef complexity, and contrasted the results against the traditional rugosity index. This study focused on damselfish to explore relationships between fishes and reef structure. Three territorial species, with contrasting trophic habits and expected use of the reef structure, were examined to infer the potential species-specific mechanisms associated with how complexity influences habitat selection. Three-dimensional reef reconstructions from photogrammetry quantified the following metrics of habitat quality: 1) visual exposure to predators and competitors, 2) density of predation refuges and 3) substrate-related food availability. These metrics explained the species distribution better than the traditional measure of rugosity, and each species responded to different complexity components. Given that a critical effect of reef degradation is loss of structure, adopting three-dimensional technologies potentially offers a new tool to both understand species-habitat association and help forecast how fishes will be affected by the flattening of reefs.

## Introduction

Structurally complex habitats provide shelter, food and other resources to a larger number of species when compared with less structurally complex habitats^1^. This relationship occurs because the three-dimensional (3D) complexity of a habitat increases the availability of refuges and barriers that fragment the living space, resulting in more heterogeneous assemblages of associated reef organisms^2^. In many ecosystems, such heterogeneity in three-dimensional structure is typically provided by foundation species, such as trees in forests^3^, corals on coral reefs^4^, and canopy-forming algae and submerged plants in riverine systems^5^. Consequently, the spatial array, growth patterns, as well as biotic and abiotic interactions of foundation species will determine the structural complexity of habitats through time^1^.

In coral reef ecosystems, both geological features and the underlying carbonate matrix, which is formed by organisms and modified over time, contribute to the structural complexity of reef habitats along with foundation species of coral^6^. These multiple scales of structure lead to more complex coral reefs hosting a greater diversity, abundance and biomass of species^4^, including fish^7^. A number of mechanisms have been proposed to explain the effect of reef structural complexity on the abundance of fish. These include mediating density-dependency through provision of niche space^8^, influencing predator-prey interactions by providing refuge for prey^9^ while increasing food availability for predators and preys^10,11^, providing nesting sites^12^, and also providing shelter from physical stress, such as water flow^13^ and ultraviolet radiation^14^. Consequently, the loss of structure on reefs as a consequence of declines in coral cover and diversity^15^ may induce a decline in the abundance of habitat-specialist species^16^ and fisheries productivity^17^. These trends are predicted to worsen under future climate scenarios^18–20^.

While an increase in total fish diversity and abundance is generally observed in structurally complex and diverse reef habitats, species-specific responses to structural complexity are less clear^4^. Previous studies have indicated variable responses to structural complexity when assessing different components of the fish community^21–23^, where both structural complexity per se and the heterogeneity or diversity of structures can play a role in habitat selectivity. Some fish species may commonly occur in heterogeneous habitats because of the benefits for feeding, mating or refuge, while other species appear to be more associated to either uniformly complex areas or even consistently low-complexity habitats^21,24,25^. For example, many species of damselfish (Pomacentridae) are known to associate with specific structural features, coral morphologies or genera^26–28^. In contrast, other Pomacentridae species associate with much less complex and homogeneous habitats dominated by macroalgae and coral rubble^29,30^. Despite these observations, a systematic understanding of which structural properties are favoured by particular fish species remains poorly understood^4^.

The problem of understanding species-specific associations to the three-dimensional reef structure largely resides in the difficulty of measuring habitat structural complexity, a concept that encompasses multiple resources across a range of scales that are differentially exploited by species^31^. On one hand, developing a single metric for structural complexity enables the development of an aggregated variable to provide a means of ranking habitats in terms of their potential contribution to biodiversity^4,32^. In particular, the rugosity index is a frequently used single metric for estimating reef structural complexity, where a tape and chain transect is used to assess the ratio between the length of the chain and the distance it covers after fitting it to the shape of the reef^33^. In response to logistical and practical limitations of measuring and dealing with multiple metrics to understand structural complexity among systems, indexes such as rugosity offer a practical solution for measuring and expressing structural complexity in a single number. On the other hand, a single metric provides little information to understand the nuances of species-specific interactions with the reef substrate. However, methods for accurately and rapidly quantifying the multiple attributes of reef structural complexity are not widely available. While previous studies have provided metrics that are useful for understanding the species-specific interactions with reef complexity, such as colony morphology, size structure of crevices, and coral height^34^, the time required underwater to quantify such attributes has constrained their use. Furthermore, potentially useful metrics, such as the field of view available to a fish in different microhabitats, are very difficult to measure *in situ*^35,36^.

Technological advances in data processing, storage, photographic sensors and computer vision are making the generation of accurate three-dimensional models of reef structure more time- and cost-effective^37–39^. Compared to traditional approaches for high-resolution bathymetrical surveys (e.g., Laser bathymetry, such as LiDAR), the more recently developed underwater photogrammetric technology offers a simpler, faster, and more affordable alternative for high-resolution topographic reconstruction^37,40,41^. Furthermore, image-based reconstruction provides two elements associated to structural complexity: (1) the structural attributes *per se*, like LiDAR, but also (2) access to the spectral attributes of the imagery, which enables more detailed observations of the ecosystem (e.g., compositional structure and seasonal or phrenological changes)^42^. Traditionally, techniques of underwater three-dimensional reconstructions have primarily been utilized for habitat classification, as well as inspections in archaeological surveys^43–45^, but recently photogrammetry from underwater footage has been explored to address ecological questions^46–48^.

We used three-dimensional reconstructions derived from stereo photogrammetry to gain insights into species-specific habitat selection by fishes on a Caribbean reef. Our objective was to develop and test novel metrics of habitat quality to elucidate the functional role of different components of the three-dimensional reef structure on the distribution of three damselfish species: *Stegastes partitus*, *Stegastes planifrons*, and *Chromis cyanea*. The three territorial damselfish species were chosen because of their (i) small home range, (ii) close affinity to reef structure, (iii) ubiquitous distribution, (iv) contrasting trophic behaviour and (v) ecological importance. Based on the life history and ecology of the chosen species, we anticipated that exposure to predators and competitors, abundance of refuges from predation, and food availability would be the key determinants of habitat selection, all being mediated by structural complexity^30,35,49^. Furthermore, we hypothesized that these key elements may be of different importance to each species because of differences in their ecology, and evaluating these differences would improve our understanding of the spatial, intra-reef distribution of the species. Finally, we hypothesized that partitioning the resources provided by structural complexity would explain the abundance of fish species more accurately than a conventional, single index of rugosity.

## Results

Coral cover at the studied sites in Glover’s Atoll, Belize, ranged from 1–18%, averaging 7%, mainly represented by corals of the genus *Orbicella*. Algae (turf and macroalgae) represented the most dominant benthic group at these sites (78% average cover), and soft-corals where the second most dominant group (12% average cover). Other benthic groups that contributed to structural complexity were sponges, but their abundance was much lower (3% average cover).

Among the studied species, *S. partitus* was the most abundant (15 ± 10 ind.25 m^−2^, mean ± std. dev.), followed by *C. cyanea* (15 ± 7 ind.25 m^−2^, mean ± std. dev.) and *S. planifrons* (3 ± 3 ind.25 m^−2^, mean ± std. dev.). The occupancy of these species also varied among grid-cells, where *C. cyanea* was the most ubiquitous species, observed in 26 out of 42 grid cells. *S. planifrons* and *S. partitus* were observed in 18 grid cells. Based on the observed spatial segregation in terms of occupied grid-cells among species, we used two approaches to explain their within-reef distribution based on structural complexity: 1) Partitioned Structural Complexity (PSC) into three metrics (viewshed, density of crevices and grazing surface area), and 2) Rugosity Index (RI).

### Comparing the capacity of metrics derived from structural complexity to explain intra-reef distribution of damselfish

**Figure 1:**
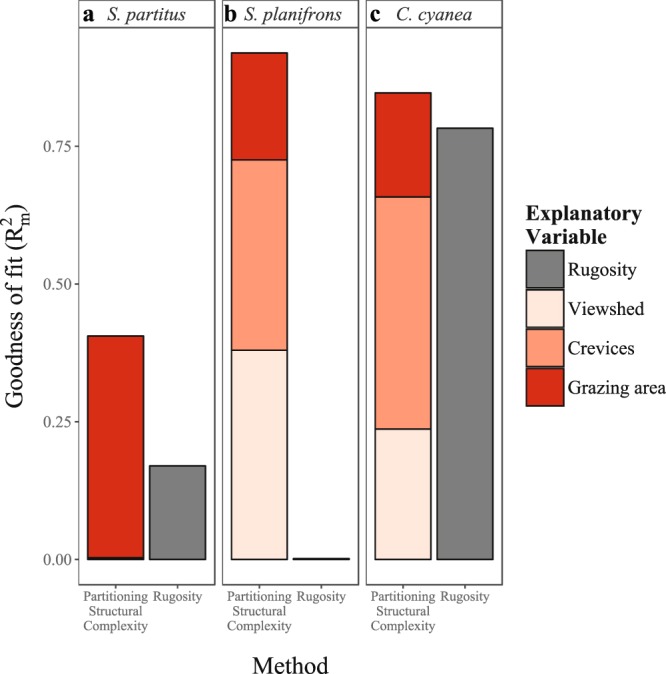
Comparison of the variance of fish abundance explained (R^2^_m_) by two different methods for each studied species (**a–c**): 1) partitioning resources provided by structural complexity (shades of red) and 2) measuring structural complexity by the rugosity index (grey). For the models using partitioned structural complexity as explanatory variables, the relative contribution of each variable to the R^2^_m_ has been segregated by calculating the relative variable importance (VIMP) and represented by different shades of red.

PSC provided a more informative model of species-specific habitat associations of damselfish than the rugosity index as a single metric of structural complexity. Regression models for the spatial distribution of each species showed that the metrics derived from photogrammetric reconstructions (viewshed, grazing surface area and density of crevices) provided a higher explanatory power (R^2^_m_) of fish abundance than the index of rugosity index alone (Fig. 1). However, the difference in explanatory power between the two methods (PSC and RI) varied among species. The most notable difference was observed for the two *Stegastes* spp, *S. partitus* and *S. planifrons*, where RI only explained 16% and 2% of the variance of fish abundance respectively. In contrast, metrics derived from structural complexity explained between 41% and 92% of the variance of the same two species respectively (Fig. 1a and b). Conversely, the difference in the explanatory power of PSC models, compared to RI models, for the planktivorous species *C. cyanea* was less contrasting. The model that included metrics partitioned from structural complexity (viewshed, grazing surface area and density of crevices) predicted the abundance of *C. cyanea* with a R^2^_m_ of 85%, in contrast to 78% obtained by modelling the abundance using the rugosity index alone (Fig. 1c).

**Figure 2:**
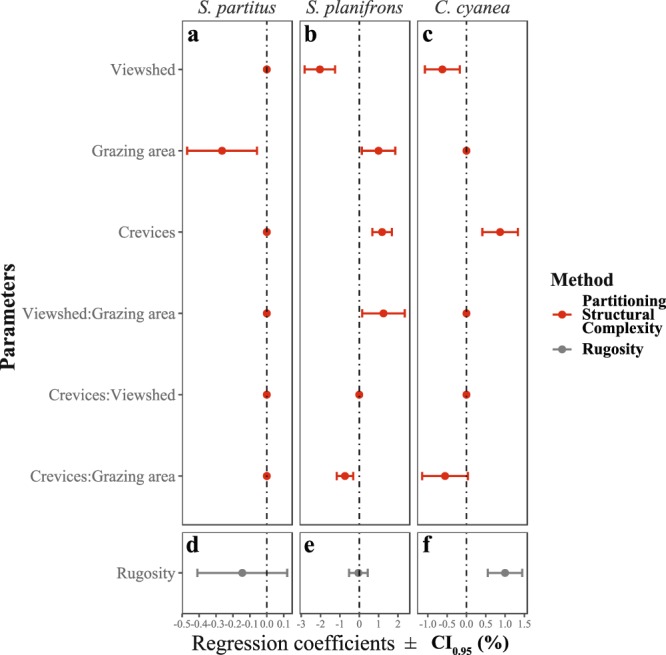
Regression coefficients for each parameter modelling the abundance of three study species using two different methods for measuring structural complexity: Partitioned Structural Complexity (PSC; red, **a–c**) and the Rugosity Index (RI; grey, **d–f**). The estimated mean of each coefficient is represented by the filled dot, while the error bars represent the 95% confidence interval.

In addition to its higher explanatory power, the PSC method provided insights into the importance of each variable in explaining the abundance of each species. The most specialised species in terms of their trophic obligation, *S. planifrons* and *C. cyanea*, showed a more complex association to structural complexity, where all three variables contributed to explaining their abundance. However, the relative contribution of these variables also varied between these two species, with viewshed having a more important contribution to the explanatory power of these models for *S. planifrons* (Variable Importance, VIMP = 41%), while the density of crevices was the most important variable for *C. cyanea* (VIMP = 50%). For *S. partitus*, grazing surface area was the only variable explaining the variance in abundance of this species (VIMP = 99%; Fig. 1a), which was the least important variable for the other two species, *S. planifrons* (VIMP = 21%, P = 0.02) and *C. cyanea* (VIMP = 22%; P = 0.07; Fig. 1b and c). Note that although grazing surface area contributed to explaining the distribution of *C. cyanea*, its contribution was not significant (P = 0.07, Fig. 2c). It is worth noticing, however, that in the particular case of *C. cyanea* the rugosity index explained more variance in the models of abundance than any PSC metric alone.

### Species-specific effects of structural complexity on fish abundance

**Figure 3:**
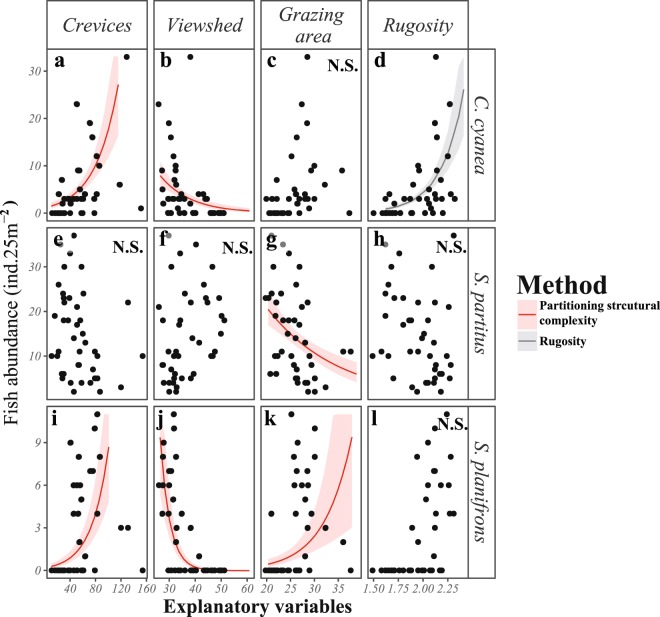
Relationship between explanatory variables and the abundance of *Chromis cyanea*, *Stegastes partitus* and *Stegastes planifrons*, using two different methods for measuring structural complexity: 1) Partitioned structural complexity (**a–c;e–g;i–k**, red) with three resource metrics (crevice density, viewshed, and grazing area) and 2) Rugosity index (**d,h** and **l**, black). Observed fish abundance (black dots) is expressed in individuals per grid-cell (ind.25 m^−^^2^). The continuous line represents the model estimates of fish abundance when varying only one parameter (in the case of partitioning structural complexity) and fixing the other parameters to the mean observed value. The shaded area represents the standard error of model predictions. “N.S.” is shown in plots where no significant effect of the variable on the fish abundance was found.

Species-specific correlations of each habitat metric from the PSC method with fish abundance were observed (Figs 2 and 3). For the planktivorous damselfish, *C. cyanea*, viewshed was negatively correlated with fish abundance (glmm, P = 0.007, Fig. 2c), while the density of crevices was positively associated to the abundance of *C. cyanea* (glmm, P = 0.0002, Fig. 2b). That is, as the reef substrate became more visually exposed (open fields, thus higher viewshed) the abundance of *C. cyanea* decreased (Fig. 3b). Conversely, as the density of crevices increased within the grid-cells, the abundance of *C. cyanea* increased (Fig. 3a). The gardening herbivorous damselfish (*S. planifrons*) exhibited a more complex association with the reef substrate as all three variables showed a significant effect on the abundance of the fish (glmm P < 0.05; Fig. 2b). The abundance of crevices and grazing substrate was positively associated to the abundance of *S. planifrons*, while viewshed showed a significant negative correlation (Fig. 2b). This is similar to *C. cyanea*, where fish abundance had a significant and positive association to more enclosed (less open, thus smaller viewshed) spaces and higher density of physical refuges or crevices (Fig. 3g,h). Grazing surface area was also positively and significantly correlated with the local abundance of *S. planifrons* (glmm P = 0.02, Figs 2b and 3i).

*S. partitus* was less influenced by the complexity metrics from either the PSC or RI methods (Fig. 1a), but showed a significant correlation with grazing surface area (glmm P = 0.01, Fig. 2a). In contrast to *S. planifrons*, this observed association was negatively related to grazing surface area (Fig. 3f). Furthermore, the standardized model coefficients showed a weaker association between the abundance of *S. partitus* and grazing surface area compared to that of *S. planifrons* (Fig. 2a).

Overall, rugosity performed worse at predicting the fish abundance for all three species, compared with metrics partitioned from structural complexity (Fig. 1). However, as a single metric, rugosity was significant in explaining the abundance of *C. cyanea* (glmm P ≪ 0.0001, Figs 2f and 3j), and accounted for a higher explanatory power (*R*^2^_*m*_) than any single PSC metric in modelling the abundance of *C. cyanea* (Fig. 1).

**Figure 4:**
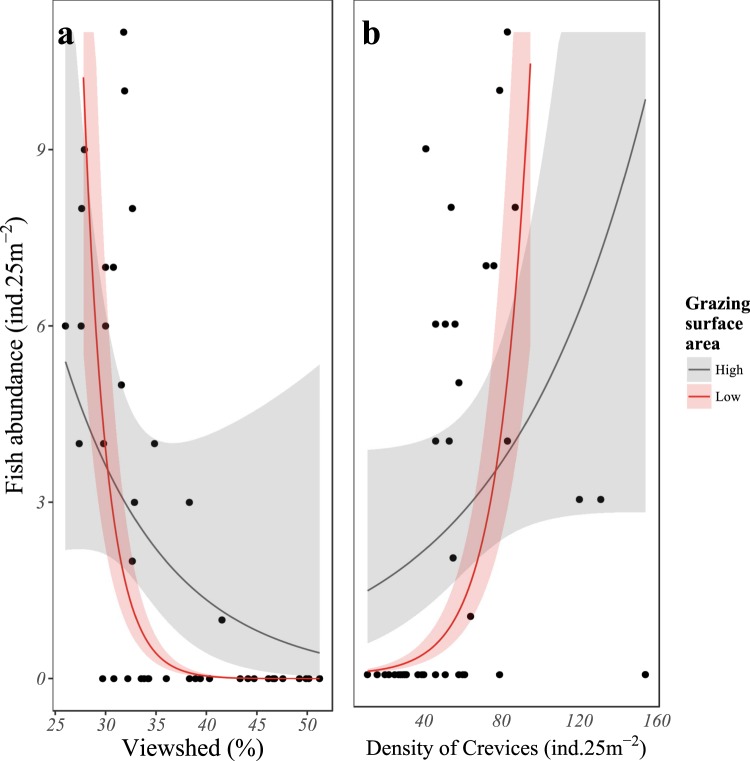
Interactive effect of: (**a**) viewshed and (**b**) density of crevices with grazing surface area on the abundance of *S. planifrons* using the method of partitioning resources from structural complexity. Model estimates of fish abundance for each grid-cell (ind.25 m^−2^) are represented by continuous lines and observed fish abundance represented by black dots. Red lines indicate predictions of fish abundance when grazing surfaced area is low (20 m^2^, mean minus one standard deviation). Black lines indicate predictions of fish abundance when grazing surface area is high (30 m^2^, mean plus one standard deviation). Shaded area represents the standard error of model predictions for high (grey) and low (red) grazing surface area.

In addition to the individual effect of PSC variables, the interaction between either viewshed or density of crevices with grazing area also had a significant effect on the abundance of *S. planifrons* (Fig. 2b). Both interactions (“viewshed: grazing area”, and “crevices: grazing area”) were represented by negative coefficients in the models (Fig. 2b), indicating that they act in different directions when predicting fish abundance. The lowest abundance of *S. planifrons* was found on reef terrains that showed either relatively high viewshed (>40%; Fig. 3g) or low density of crevices (<80 crevices.25 m^−2^; Fig. 3h). In contrast, the largest abundance of *S. planifrons* occurred in areas with either low viewshed or high density of crevices (Fig. 3). Grazing surface area, on the other hand, weakened this relationship. In habitats with low grazing surface area, the relationship with viewshed (Fig. 4a) or density of crevices (Fig. 4b) was the strongest, but in habitats with high grazing area, the relationship of fish abundance with viewshed or density of crevices became more variable and less strong (Fig. 4). The interaction between viewshed and crevices, however, was not significant for any of the species.

## Discussion

Using novel technologies for measuring the three-dimensional complexity of reef structures on the Mesoamerican Barrier Reef, this study explored the relationship between substrate structure and the within-reef distribution of three damselfish species. By partitioning the relative importance of structure on food availability (grazing surface area) and predator risk and spatial competition (viewshed and density of crevices), we evaluated the link between resources provided by structural complexity and the abundance of each fish species. The species-specific relationships with each structural factor varied from weak to strong, and demonstrating that the new insights can be obtained with the use of three-dimensional methodologies that can quantify multiple aspects of complexity, such as viewshed, grazing area and density of crevices. In addition to improving our understanding of the factors controlling the abundance of each damselfish species, the resulting models also provided more explanatory power than methods using the traditional rugosity index. The rugosity index, however, was a better single predictor of the abundance of *C. cyanea* because it explained a higher percentage of the variance than any single PSC metric alone. Having said that, insights on the factors controlling this association would be difficult to disentangle using this single metric of structural complexity, which is possible by partitioning the resources provided by structural complexity *sensu* the method described in this study.

The interaction between predator and prey is arguably the one most influential processes driving the distribution and abundance of fish species within coral reef habitats^50,51^. As a result, specific behaviours have evolved that are associated with avoiding predation risk in the case of prey, and maximising hunting success in the case of predator^52,53^. Such behaviours include reproduction, feeding, territorialism, aggregation and competition, and can be strongly mediated by structural complexity through resource partitioning^9,11,35,36^. The role of structural complexity in influencing habitat selection by reef dwelling species is hard to disentangle from correlations of field observations of fish abundance with summarising metrics of reef structural complexity (e.g., rugosity indices). While experimental manipulation has demonstrated species-specific association of fish to reef three-dimensional structure^35,36^, summarising indices of rugosity cannot provide direct insights on species associations because resources are invariably exploited differentially by each particular species^49^. Here, we used advances in underwater photogrammetry to expand the investigation of the role of physical structure in mediating associations of fish to reef habitats. This was done by quantifying key attributes that, supported by experimental observations, were directly related to the effect of predator-prey interactions and food availability on habitat quality.

Overall, these results show that attributes associated with predation avoidance were the most important correlates for the distribution of two trophically specialised species of damselfish. The contribution of microhabitats, created by structural complexity, to offer shelter from predation was evaluated by: 1) deterring visual encounters with predators (viewshed) and 2) providing physical refuges to predator attacks (crevices). Specialised prey species can be affected by predation through changes in prey abundance or changes in their behaviour in response to predation risk. In the case of resident predators, such as groupers, snappers and other coral reef fish predators^50^, all prey may take refuge in a protected microhabitat and thus converge in resource use^50,54^, thus explaining the patterns observed in this study.

### Structural complexity and the biology of damselfishes

The within-reef distribution of damselfish species revealed contrasting responses explained by different structural attributes, as expected given the difference in habitat use among the studied species.

The observed relationships between PSC metrics and the abundance of fish agrees with the expected habitat associations, based on the trophic ecology of each species. *C. cyanea* is a specialised planktivorous species^35^, therefore we expected a weak association to the availability of grazing area. This species is often observed on top of coral colonies which have branching morphology (e.g., *Orbicella annularis*, *Acropora cervicornis*) because the height of the colonies offer access to the plankton suspended in the water column, while the morphology of the coral colony offers immediate access to refuge from predators^55^. *S. planifrons* is an algae farmer and herbivore, which commonly associates with to coral colonies that provide a high density of crevices^34^, and therefore the availability of grazing substrate and crevices were expected to influence the local abundance of this species. *S. partitus* association with grazing area was expected to be weak because this is one of the few *Stegastes* species that commonly feeds on benthic plankton and less so on filamentous algae^56,57^.

Viewshed was a strong predictor of the abundance of two species, *S. planifrons* and *C. cyanea*. This result is consistent with previous observations of damselfish species, where structural complexity appeared to mediate predation risk by offering visual escape from predators^8,28,57^. For example, field correlative observations as well as experimental manipulation of field of view in a patch reef showed that the abundance and territory size of *C. cyanea* linearly increased as the reef configuration became more visually enclosed^35^ (lower viewshed in this study). While *Chromis* spp aggregation is an effective anti-predator mechanism, the restricted visual access provided by structural complexity may contribute to avoiding encounters with predators^55,58,59^.

While reduced visual exposure of a terrain offers a potentially safer environment to some species in terms of reducing potential interactions with predators and competitors, crevices offer immediate and physical refugia to predator attacks. Crevices are created by erosion of the reef matrix, spatial configuration of foundation species or intrinsic morphological traits of these species (e.g., branches of *Acropora* spp and ramets of *O. annularis*). They offer safe refuges from predation and therefore a highly valuable resource from small prey species. The abundance of *S. planifrons* and *C. cyanea* was positively correlated with the number of crevices available, as previously reported^35,49^, and this trend is likely to be true for other highly territorial and reef-associated pomacentrids, such as *S. adustus*^34,56,60^.

### Interaction between food availability and predation risk

Different behavioural responses to predation risk can be expected among prey depending on the trade-off between risking mortality *versus* maximising other aspects of fitness^61^ (e.g., foraging, nesting care or reproductive success, competition). Four contrasting behaviours can be summarised^61^: 1) risk reckless, when prey expose themselves to full risk in order to maximise other attributes of fitness, 2) risk avoidance, when the prey actively look for habitats that minimise predation risk despite the implications of limited resources (e.g., food, mating), 3) risk adjusting, when prey respond to an increase in predation hazard by proportionately reducing exploitation of food, irrespective of the amount or quality of food available and 4) risk balancing, when prey assume the risk of predation when it is counter balanced by the rewards in foraging efficiency^61^.

*S. planifrons* appears to exhibit a risk balancing behaviour. Factors associated with predation risk avoidance (viewshed and density of crevices) are strongly correlated to the distribution of this species, while a higher availability of food resources (grazing surface area) weakens the importance of habitat attributes and predator avoidance. This strategy corresponds to that formulated by Werner & Gilliam^62^, which was later supported by manipulative experiments of food supply and predation shelter on gobies^63^, whereby an increase in predation risk was compensated for by an increase in feeding opportunity. Therefore, while generally choosing habitats that provide more shelter from predation risk (viewshed or density of crevices), some individuals of *S. planifrons* occupy riskier habitats when food resources are more abundant. It is expected that these strategies among prey species will vary over time and space according to extrinsic condition and ontogenetic shifts (e.g., abundance of predators over time, and the life-stage of the prey individuals)^61,62^. Interactions amongst competition, body size, daily cycles and predation risk can also lead to counterintuitive outcomes on habitat occupancy by prey species^62^. A recent review of the risk allocation hypothesis suggests that prey are not necessarily ‘living on the edge’, in terms of meeting their energy demands^64^. While species can reduce their foraging activities during high predation risk, energy intake must be compensated during low risk situations^64^, suggesting a more adaptive risk behaviour^61^. However, current literature only provide mixed support for these models^64^. The temporal variation of the prey response to predator cues and the spatial scales at which risk allocation influence microhabitat selection are not yet fully understood^64^. Further manipulative experiments, across a range of temporal and spatial scales, will be required to confirm our observations.

### Other drivers and limitations associated with structural complexity

In contrast to the observed negative effect of increased viewshed on the abundance of some species, enclosed habitats may also be considered a riskier situation for other prey species. For example, by experimentally reducing the visual field around males of *S. partitus*, courting rates and the distance they ventured away from the nest for feeding consistently decreased^36^. Consequently, it appears that limited visual fields can present a riskier situation than a clearly visible predator to smaller species, such as *S. partitus*^56^. This relationship was not found in the present study, perhaps indicating that other factors may play a more important role in driving the intra-habitat distribution of *S. partitus*. At this point, it is important to mention that, given the spatially heterogeneous distribution of *S. partitus*, a higher replication of the survey plots within reefs could better capture the variability of the association of *S. partitus* to the reef substrate, while accounting for other drivers such as predator abundance and density of competitor species (*sensu*^34^).

Limitations associated with the three-dimensional reconstructions could explain the low explanatory power in the distribution of *S. partitus* and the lack of correlations with viewshed and the number of crevices. Firstly, image-based 3D reconstructions are not able to represent moving objects, and therefore large sea fans and other soft corals are excluded from our metrics, despite providing structural complexity^65^ and influencing fish communities in complex ways^66^. For example, sea fans can contribute to reducing the field of view of reef organisms, and *S. partitus* is less abundant as the abundance of soft-corals increases due to an increase in the uncertainty to predation^36^. Thus, incorporating soft corals into metrics of structural complexity may increase the explanatory power of statistical models. Evolving approaches in photogrammetry reconstructions may overcome the limitation of considering flexible benthos in three-dimensional reconstructions^67^. Secondly, small-scale structure could not be resolved because of the low-resolution cameras used. The scale at which structural complexity influences the abundance of species is proportional to their size^50,68^. Given that *S. partitus* associates to coral rubble patches^36^, small-scale crevices provided by rubble can offer physical shelter from predation, but this could not be resolved from our reconstructions, mainly because of their spatial accuracy (centimetres)^44^. Higher resolution sensors and more detailed photography of coral colonies can achieve a much higher resolution, even similar to laser scanners^37^, and may help to resolve this technical limitation and allow for the detection and mapping of small-scale refuges is possible. It is important to note however, that species-specific associations to reef substrate may operate at different spatial scales^49^, and further investigation is needed to understand how spatial accuracy of three-dimensional reconstructions would influence detectability.

Inter- and intra-specific interactions such as competition and hierarchical social structure can also influence the abundance and distribution of fish species^69^, in particular for *S. partitus*^34^. While structural complexity can mediate these interactions by partitioning resources through habitat heterogeneity^21,70^, density-dependent mortality and recruitment are also strong drivers of fish abundance in space-limited habitats, such as coral reefs^58,71^. *S. partitus* has a more complex social structure compared to the other two species, where hierarchies play an important role in microhabitat partitioning^72–74^. This social structure is size-dependent, where larger alpha and beta males (7–8 cm) control the distribution of sub-ordinated smaller individuals due to high levels of intra-specific competition. Therefore, different individuals within the social hierarchy may be expected to occupy contrasting habitat types^72^. Larger and dominant individuals are expected to be associated with enclosed habitats with a high density of refuges (lower viewshed and high density of crevices), while the smaller individuals are forced to suboptimal territories where the microstructure provided by coral rubble can offer shelter from predation. In addition, interspecific competition with more aggressive species, such as *S. planifrons* can limit the abundance and spatial distribution of *S. partitus*^30,75^. Our focus was on contrasting different metrics of complexity, but considering the size structure and the spatial overlapping of potential competitors may help to better understand the drivers of the distribution of *S. partitus* on reefs.

### Role of three-dimensional technology in extending our ecological knowledge

In addition to complementing generic metrics of spatial heterogeneity of reef structure, three-dimensional reconstructions of the reef habitat provide the opportunity to quantify specific resources associated with habitat quality that drive species-specific responses in fish abundance. Fast processing and large-scale surveys are favourable attributes of underwater photogrammetry when calculating summary indices, such as the rugosity index, and overcome the need for a large number of chain transects while underwater^4^. This technology, brought up by computer vision through image-based reconstructions, offers a yet underexplored alternative to rapid surveys of large areas, while providing the means to quantify an increasingly novel set of metrics to better understand patterns and processes otherwise limited by the logistical constrains of underwater work.

Applications of the framework described in this study are not limited to correlational observations of fish abundance and structural complexity and we anticipate that this framework can be applied to other ecological studies. For example, using experimental manipulations of reef structure or predator abundance (*sensu*^35,76^) and systematic field observations of fish demographic and behavioural traits (*sensu*^50,77^), viewshed analyses of the reef topography may help to improve our knowledge of the role of predatory cues in driving population dynamics and functional processes in prey species (e.g., herbivory, energy transference, productivity, biodiversity). Alternatively, metrics derived from three-dimensional technologies may also facilitate comparisons between artificial and natural reefs. Along with the increasing interest in artificial structures for protecting coastal populations and providing alternative energy sources, interest in designing marine structures that sustain vital ecosystems services is also growing^78,79^. Evaluating the performance and needed attributes of artificial structures to resemble natural reefs is not straight forward, resulting in a paucity of unequivocal evidence that artificial reefs fulfil their intended objectives^80^. Reconstructing the tree-dimensional structure of artificial structure and natural coral reefs could help to derive comparative metrics that allow us to understand not only the effects of the physical properties of artificial habitats on the colonizing biota, but also their effects on processes such as predation and competition, which will ultimately improve our understanding of the performance of artificial reefs^79^.

In this study, we used a standardised and calibrated method for three-dimensional reconstructions, close-range photogrammetry from stereo imagery^44^. While one of the powerful advantages of this approach is that it produces scaled reconstructions without the constant need for camera calibration from reference scales in the field, it is generally more expensive than using monocular and off-the-shelf camera equipment. Novel applications of monocular reconstructions using Structure from Motion (SfM) algorithms are proven to be more attainable methods for general use while generating high-precision in three-dimensional reconstructions^37,38,81^. Given that data outputs from either monocular and stereo reconstructions are the same (3D point cloud, surface mesh and photomosaic), applications of the framework described here are equally transferable to reconstructions generated from cheaper, standard and off-the-shelf monocular cameras and software (e.g., GoPro cameras and Photoscan software for reconstructions)^81^.

Greater explanatory power or goodness-of-fit in the method partitioning structural complexity could be higher than when considering only one metric (rugosity), because there are more covariates to explain the behaviour of the response variable. However, in addition to outperforming traditional metrics, partitioning the resources provided by structural complexity offers more informative data to better understand species-specific fish associations to reef substrates. Furthermore, our models were carefully selected to favour the most parsimonious model, to avoid overfitting or losing information. This comparative approach outweighs the effect of multiple covariates in explaining the response variable. Results simply tell us that segregating the resources provided by structural complexity into metrics that are relevant to the biology of the studies species is, in the end, more informative than rugosity when explaining the spatial distribution of damselfish within a reef. Despite being the most commonly used metric, the rugosity index is only one of an evolving suit of metrics that describe structural complexity in coral reefs^4^. Counting crevices, measuring the morphology and assessing spatial distribution of coral colonies and other organisms (e.g., soft corals), can help explaining the distribution of fish species within a reef^34,36,49^, in a similar fashion than this study. However, measuring these metrics is time consuming, and sampling effort is constrained by the limited field time available for underwater surveys. Our method, on the other hand, is faster and has lower field time requirements because once images are collected underwater the multiple structural complexity measurements can be derived from the computer.

As coral reef ecosystems experience accelerated rates of decline under recurrent and broad-scale disturbances^82^, important loses in structural complexity have been observed region-wide^15^. Global disturbances under a changing climate are expected to accelerate erosion and depressed calcification, altering the permanence of essential framework habitat^18,83,84^. The interactive effects of losing structural complexity and selective overfishing of predators, and potentially meso-predator release, may lead to unexpected outcomes of species dominance and localized extinctions^18,85^. Improved understanding of how different habitat configurations, varying in complexity, composition and coral cover will affect the affinity of reef fishes to coral reefs under changing scenarios will better inform spatial prioritization for conservation. We believe that adopting three-dimensional technologies in coral reef studies will contribute to better understanding the effects of reefs becoming flatter by both allowing considerations of structure at larger spatial scales and account for the natural spatial heterogeneity of natural systems, and disentangling the nuances of specific-specific association with reef structure.

## Methods

### Study area

**Figure 5:**
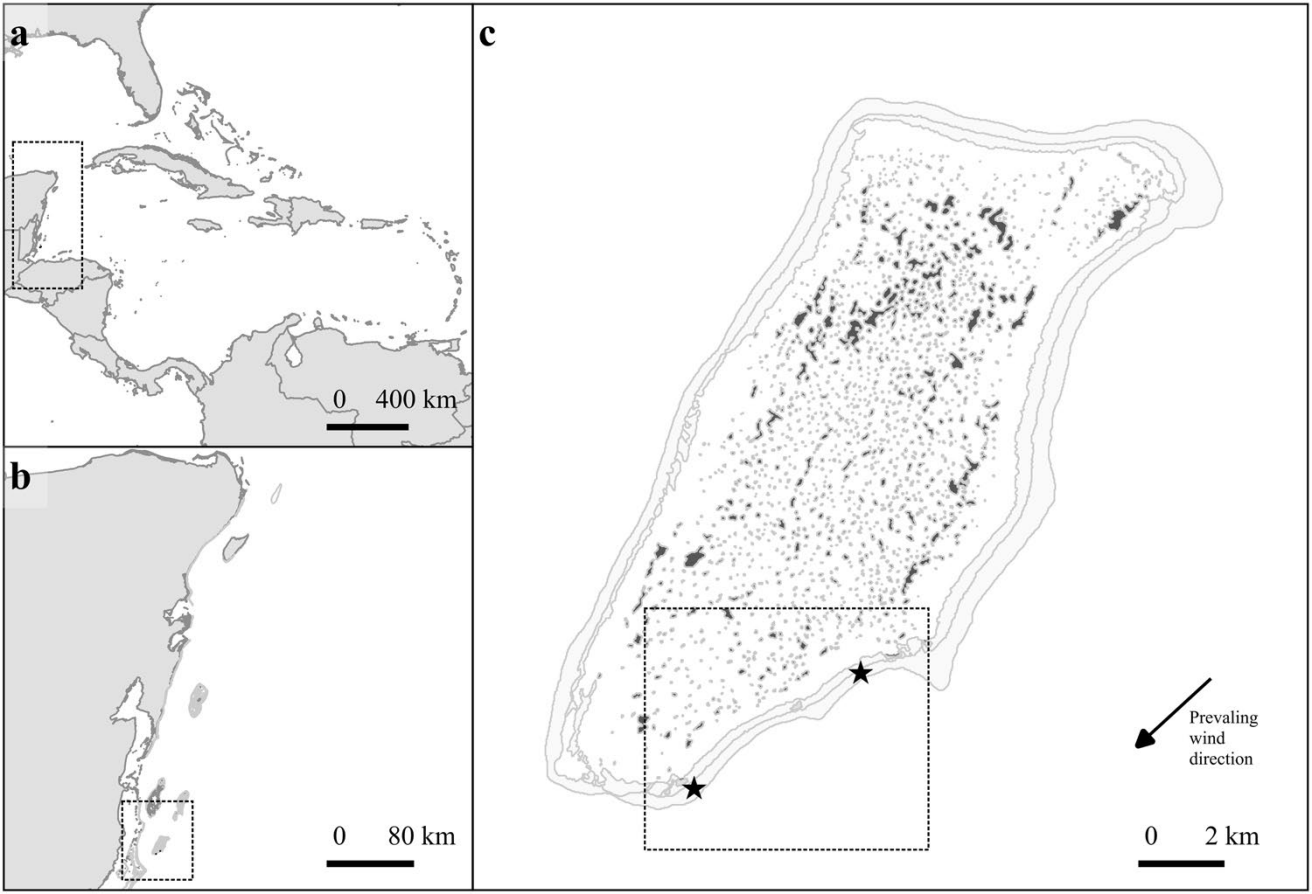
General location and study sites: (**a**) Caribbean region, (**b**) Mesoamerican Barrier Reef System, (**c**) Glover’s Reef Atoll, Belize. Black star symbols show the location of study sites. Map produced in QGIS 2.18 (www.qgis.org) using the following data sources: National Geospatial-Intelligence Agency (base map, World Vector Shoreline Plus, 2004. http://shoreline.noaa.gov/data/datasheets/wvs.html)and UNEP-WCMC *et al*. 2010 (coral reefs^102^). The location of survey sites was obtained from the present study. Data sources are open access under the Creative Commons License (CC BY 4.0).

This study was conducted on *Orbicella*-dominated reef habitats, previously known as *Montastraea* reefs^86^, located on the windward side of Glover’s Atoll within the Belizean section of the Mesoamerican Barrier Reef System (Fig. 5). This reef habitat is common on sheltered to moderately exposed forereefs throughout the Caribbean^87,88^ and is typically characterized by being dominated by *O. annularis* and *O. faveolata*. As the main ecosystem engineers, these two species create habitats that vary in three-dimensional structural complexity at different scales: (1) small scale (centimetres) determined by crevices within coral ramets, (2) medium scale (centimetres to metres) provided by the morphology of coral colonies and their epibionts (e.g., soft-corals, other hard coral species) and (3) mesoscale (10–100 s of metres) consisting of the spatial distribution of coral colonies and interspersed microhabitats^49^. While reef architectural complexity tend to increase with coral cover, it is generally highest in habitats dominated by the reef-building genera *Orbicella*^89^. Despite the commonly observed low coral cover levels in the Caribbean region, standing colonies of this genera form structurally complex habitats associated to a high diversity of species^87,90^. A spatially nested sampling design was used for our study at two reef sites separated by seven kilometres, both at 10−12 m depth. At each site, three 15 × 15 m (225 m^2^) plots were haphazardly chosen and subsequently divided into grids of nine 5 × 5 m (25 m^2^) grid-cells. On average, the distance among plots within a site ranged between five and ten metres, approximately.

### Three-dimensional reconstruction of the reef structure

A stereo-camera system developed by the Australian Centre for Field Robotics (ACFR)^39,44,91^ was used to collect consecutive stereo imagery at a frequency rate of 2 Hz and a resolution of 2 megapixels across the area delineated for each survey plot. Imagery was collected following a “lawn-mowing” pattern designed to maximise image overlapping along and across the track and enabling processing imagery into visual three-dimensional reconstructions without gaps in coverage^44^. In addition to the imagery, co-registered sensor data including compass heading, GPS location, depth and altitude were collected and used to provide accurately georeferenced pose estimates using Simultaneous Localisation and Mapping (SLAM)^92^. The accurate pose estimates, along with the stereo-image pairs were then fed through a stereo photogrammetry pipeline^93^ and were used to recreate the three-dimensional reef structures within and around the plots (two-metre buffer zone).

A three-dimensional composite mesh (Triangular Irregular Network) of the survey area and a photomosaic of the area orthographically projected using the 3D composite mesh and camera poses were generated using a standard stereo photogrammetry architecture of algorithms designed by the ACFR to estimate camera poses^44,94^.

### Fish census and chosen species

Stationary observations were made from about two metres from the reef substrate and during five minutes within each grid-cell (5 × 5 m area) of every plot to count all fish species. Observations were timed to 5 min to maximise detection of all fish within each grid cell, while carefully examining the area to avoid overestimation of fish abundance. Three territorial damselfish species were chosen for this study given their small home range, known affinity to reef structure, cosmopolitan distribution, and contrasting use of the reef habitat based on their trophic classification, functional role and territorial behaviour (Table 1).

**Table 1:** Life history and ecological traits of model species.

Species	Trophic classification	Aggregation	Behaviour	Territory size (m^2^)	Reaction distance (m)	Average observed size (cm)	Habitat use	References
*Stegastes planifrons*	Herbivorous	Solitary	Aggressive	2.5	0.5	3	Farm gardens of turf algae. Strongly associated to *Orbicella* colonies.	60,103–105
*Stegastes partitus*	Omnivorous	Solitary	Aggressive	4-5	—	5	Associated to rubble areas. Mortality of individuals higher on boulder coral habitats than in rubble habitats	36,56,57,76
*Chromis cyanea*	Planktivorous	Gregarious	Passive	3-15	1	4	Abundant on top of *Orbicella* colonies. Retreat to crevices under coral colonies when frightened.	35,55

### Partitioning structural complexity into functional components

We used a number of characteristics from the three-dimensional reconstructions (3D composite mesh and photomosaic) to calculate four different metrics for each plot as proxies for the following attributes: 1) viewshed, 2) density of crevices, 3) grazing surface area and 4) reef rugosity. In this study, the capacity of these metrics to explain the abundance of each damselfish species was evaluated by two methods: 1) A novel approach whereby the resources provided by structural complexity were portioned into three key attributes for these species (viewshed, density of crevices and grazing surface area); and 2) A traditional approach where the abundance of the fish was investigated as a function of a single metric of structural complexity: the rugosity index.

#### Viewshed

**Figure 6:**
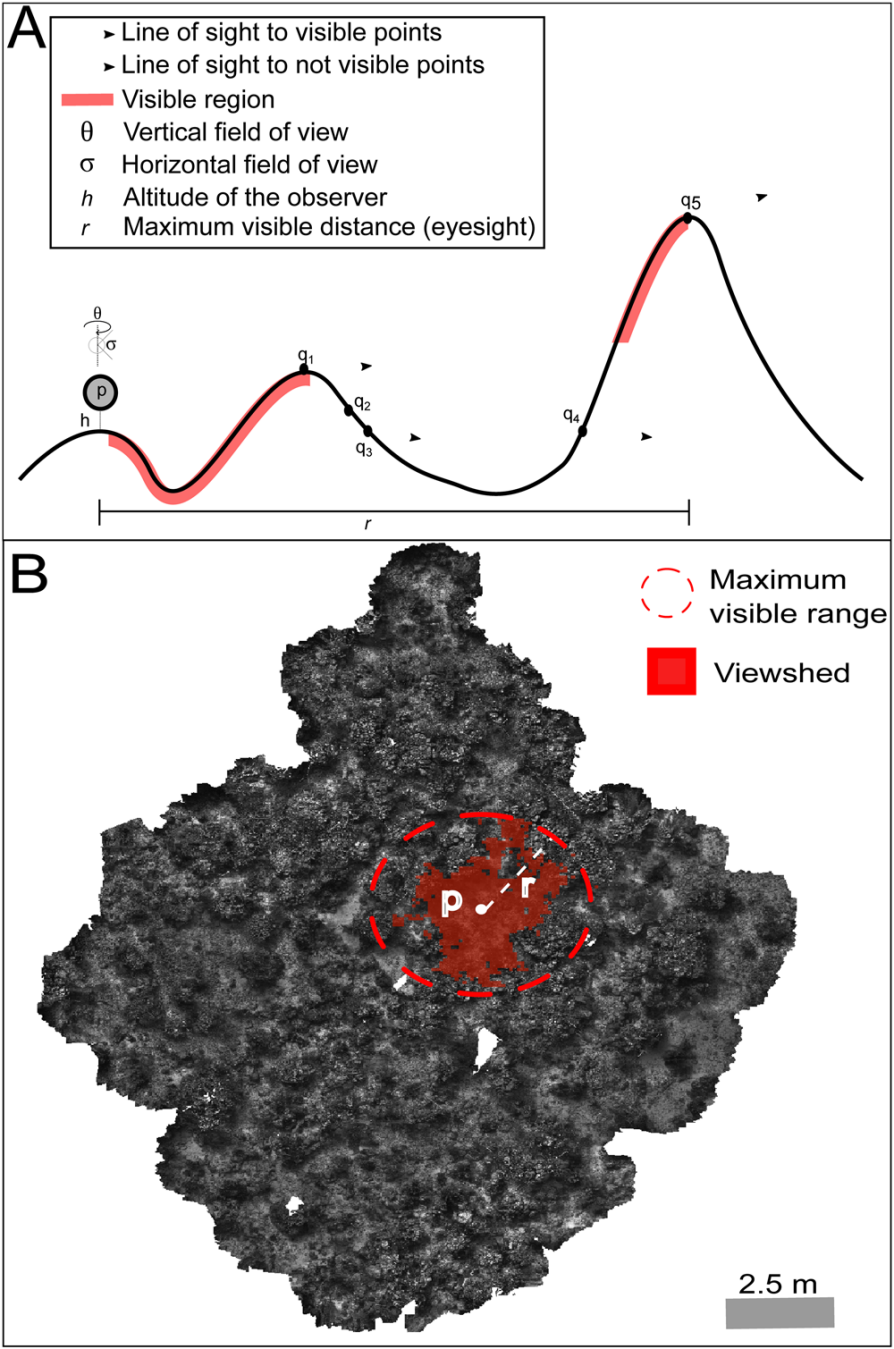
Viewshed analysis diagrams: (**A**) point visibility from observer p at altitude h to illustrate how visible points on the terrain determined (q_1_ and q_5_) to estimate the visible area. Each observer is assigned maximum field of view in the vertical and horizontal profile (${\boldsymbol{\theta }}\,$and ${\boldsymbol{\sigma }}$). (**B**) Area visible from a given location (p) on the photomosaic. Using the Triangular Irregular Network (mesh) derived from 3D reconstructions, the visible area is then calculated for randomly laid points on the terrain. Exposure to predators and competitors is calculated as a ratio of the viewshed by the potential visible area (assuming no terrain interference).

Generally, viewshed is defined as the proportion of terrain that is visible from a given location and is commonly used in geographic planning for the optimization of the location of radars, fire towers, and communication towers in order to maximise coverage^95^. Given that exposure to predators and competitors relates to the degree of openness of a terrain to predators or competitors, here we calculated viewshed to estimate the degree of openness of a section of the reef, based on hypothetical fish positioned in a given location. For any location on the terrain, viewshed analysis identifies all the points in the terrain (*q)* that can be seen by the observer (*p*), given a set of parameters intrinsic to the observer: detection range, horizontal and vertical field of view (*r, θ* and σ respectively; Fig. 6A). Using the Triangular Irregular Network (TIN or mesh) derived from the 3D reconstruction of the reef, viewshed analysis calculate the visible area (viewshed) by adding the individual area of each the points (*A*) on the terrain that are visible by the observer (*v*; equation 2, Fig. 6B).

1 $${\nu }_{(P,r,\theta ,\sigma )}=\{q\in \tau |{d}_{(P,r,\theta ,\sigma )}\le r\,{\rm{and}}\,{\rm{q}}\,{\rm{is}}\,{\rm{visible}}\,{\rm{to}}\,{\rm{p}}\}$$

2 $$vs={\sum }^{}A({v}_{(p,r,\theta ,\sigma )})$$

In order to calculate the viewshed of the terrain for each plot, simulated observers (fish) were randomly located within each plot. For each hypothetical observer, we systematically calculated the viewshed as the effective visible area relative to the potential visible area (i.e., the eyesight without terrain obstruction).

For simplicity, vertical and horizontal field of views were set to their maximum values (180° and 360°, respectively), thus, the viewshed is determined by the complexity of the terrain and not by the combination of terrain complexity and the field of view of the observer. Based on the reaction distance to predators and territory intruders, as well as the average territory size measured for these species (Table 1), we set the detection range (*r*) to 3 m for all species. The height (*h*) of the observer and target points on the terrain was set to 10 cm. For each plot, 900 simulations of fish were run at random locations within the terrain (100 fish per grid-cell). Viewshed for each point was calculated in Python (v 2.7.11, Python Software Foundation, Delaware, USA), using the viewshed function within the arcpy module (Environmental Systems Research Institute, California, USA), and averaged within each grid-cell.

#### Density of crevices

This metric quantifies the availability of immediate physical refuges (i.e., hiding spaces) from predation. A physical refuge was defined as any crevice in the terrain of more than 10 cm in width, which corresponds roughly to the maximum size of counted fish in this study. We estimated the availability of predation refugia within a gird-cell by counting the density of crevices directly from the scaled photographic mosaic.

#### Grazing surface area

As the surface area increases, it is expected that the availability of substrate-associated food sources, such as algae, also increases. However, the relationship between surface area and food availability also depends on the coverage of sessile organisms that area not consumed (e.g., corals, sponges, or soft-corals). We hypothesized that the grazing surface area influences the intra-reef distribution of *S. planifrons* and *S. partitus*, which are respectively classified as strict and facultative herbivores (Table 1).

Grazing surface area was quantified by outlining turf algae in the photographic mosaic of each grid-cell, and then translating this to surface area by overlaying these delineated polygons onto the three-dimensional terrain model derived from the reconstruction.

#### Rugosity index

The rugosity index is a measure of the deformation of a surface relative to its planar projection, and it is a common metric used to characterize the architecture of reef habitats^4^, where a value of 1 depicts a perfectly flat surface and the index increases with the complexity of surface convolutions. While rugosity is typically measured using the chain-and-tape method^33^, but it can be calculated with precision from 3D reconstructions of the seafloor^37,44,48^.

Rugosity $({f}_{r})$ was calculated as the ratio of the surface area of the convoluted terrain (${A}_{r}$) with its projected geometric surface area (${A}_{g},$ Equation 3)^37,44^. For this calculation, we used a window size of 25 m^2^, which is comparable to the area used for viewshed calculations and the territory sizes among species (Table 1). Multiple window sizes we are also tested (0.25, 1 and 25 m^2^), but the latter showed the highest correlation to the abundance of all three fish-species.

3 $${f}_{r}=\frac{{A}_{r}}{{A}_{g}}$$

### Data analysis

Relationships among fish abundance and the four metrics of structural complexity were evaluated using generalised mixed-effect models (glmm). We built models for individual fish species under two contrasting assumptions and methodologies: 1) partitioning the global effect of structural complexity into three components: viewshed, crevice density and grazing surface area; or 2) measuring structural complexity defined by the rugosity index. Models including all three variables partitioned from structural complexity included their individual effect as well as the second-order interactions amongst these variables (“grazing area: viewshed”, “grazing area: crevices” and “crevices: viewshed”). Reef site and plot ID were modelled within the random effects of the model to account for the spatially nested sampling design. Poisson or negative binomial link functions were used to parameterise the over-dispersion of model residuals given the nature of the count data. Zero-inflated data was accounted into the model by splitting the data into presence - absence and abundance within an Automatic Differentiation (AD) model building framework, using the glmmadmb package^96,97^ in R. Model simplification was performed by computing all possible combinations of explanatory variables, then selecting the most parsimonious model based on the Akaike Information Criterion^98^, using the MuMIn package in R (see Supplementary Tables S1–3 online for the model simplification table for each species).

The explanatory power of the three metrics of structural complexity in explaining the spatial distribution of each fish species was compared to a model whereby only rugosity was included as predictor. R^2^ values, calculated as the proportion of variance explained by a model (pseudo-R^2^ for generalized mixed-effect regressions)^99^, were used as a metric of goodness-of-fit to compare models among species. To ease its interpretation, the fixed effect variables were centred to zero using their mean values^100^. Since the random components ($\gamma$) were the same across all models, and because we were interested in comparing the influence of the fixed effects, here we report the marginal R^2^ (${R}_{m}^{2}$, equation 4). This equation considers the variance of the fixed effect (${\sigma }_{f}^{2}$), random effects (${\sigma }_{\gamma }^{2}$), error (${\sigma }_{e}^{2}$), as well as the intercept of the regression for the distribution-specific variance (${\beta }_{0}$), to estimate the proportion of the total variance attributed to the fixed effects^99^.

4 $${R}_{m}^{2}=\,\frac{{\sigma }_{f}^{2}}{{\sigma }_{f}^{2}\,+\,{\sigma }_{\gamma }^{2}\,+\,{\sigma }_{e}^{2}\,+\,ln(1/exp({\beta }_{0})\,+1)}$$

Given that the models using the method of partitioning structural complexity were comprised of multiple variables, here we calculated the contribution of each variable, where included, to the overall ${R}_{m}^{2}$. This contribution is here defined as the Variable Importance (VIMP) and represents the expected proportional contribution to the variance explained by the model (${R}_{m}^{2}$). When significant in the models, the relative importance of each predictor variable was evaluated using a perturbation analysis^101^, by systematically introducing random noise in each variable and evaluating its impact in the overall goodness of fit of the model (${R}_{m}^{2}$). For this approach, a random distribution of data points within the limits of each variable individually to eliminate their effect on the response variable, while maintaining the structure of the model intact. Perturbation analysis was conducted through 100 iterations for each parameter independently and the median ${R}_{m}^{2}$ calculated for each model was used to contrast against the ${R}_{m}^{2}$ obtained without perturbation ($\Delta {R}_{i}^{2}$). The Variable importance (VIMP) is then defined as the difference between perturbed and unperturbed ${R}_{m}^{2}$ relative to the sum of $\Delta {R}_{i}^{2}$ for each perturbed variable (*i*).

5 $$\Delta {R}_{i}^{2}=|\overline{{R}_{(m)l}^{2}}-{R}_{m}^{2}|$$

6 $$VIM{P}_{i}=\frac{\Delta {R}_{i}^{2}}{{\sum }^{}\Delta {R}_{i}^{2}}$$

### Data availability

Data, methodological protocols and model selection tables can be freely accessed from the following online repository: https://github.com/mgonzalezrivero/fish-structural_complexity.git.

## Electronic supplementary material

Model simplification and selection for explaining the abundance of each study species
