# Supplementary material for: Linking fishes to multiple metrics of coral reef structural complexity using three-dimensional technology
Source: Sci Rep. 2017 Oct 25;7:13965. doi: 10.1038/s41598-017-14272-5 (PMC5656654; doi:10.1038/s41598-017-14272-5)
Supplement: Supplementary file 1 — Model simplification and selection for explaining the abundance of each study species [file 41598_2017_14272_MOESM1_ESM.pdf]

Linking fishes to multiple metrics of coral reef structural complexity using three-dimensional technology

González-Rivero M [1,2*], *Harborne A R* [2,3,5], *Herrera-Reveles A* [4], *Bozec Y M* [5,2], *Rogers A* [5], *Friedman A* [6,7], *Ganase A* [1,2,5] *and Hoegh-Guldberg O* [1,2,5]

[1] The Global Change Institute, The University of Queensland, St Lucia, Queensland, Australia 4072
[2] Australian Research Council Centre of Excellence for Coral Reef Studies, The University of Queensland, St Lucia, Queensland, Australia 4072
[3] Department of Biological Sciences, Florida International University, North Miami, Florida 33181
[4] Instituto de Zoología y Ecología Tropical, Universidad Central de Venezuela. Caracas, Distrito Capital, Venezuela 1051
[5] School of Biological Sciences, The University of Queensland, St Lucia, Queensland, Australia 4072
[6] Greybits Engineering, Sydney, New South Wales, Australia 2029
[7] The Australian Centre for Field Robotics, University of Sydney, New South Wales, Australia 2006

[*] correspondence author, e-mail: m.gonzalezrivero@uq.edu.au

Supplementary Information 1. Model simplification and selection for explaining the abundance of each study species

Supplementary Table S1. Model selection table for *Chromis cyanea*. The table show the estimated coefficients, degrees of freedom, LogLik, and Akaike Information criterion for each model where the combination of explanatory variables was evaluated during model simplification. The models are ordered by AIC values, where the selected model is the one in the first row, with AIC values closest to zero, delta AIC zero and maximum AIC weight, when compared against all other model combinations. "NA" values in the Model coefficients columns indicate that that specific variable or interaction of variables was not included in the model as part of the iterations for model simplification.

Model ID	Model coefficients for selected variables							Degrees of Freedom (df)	logLik	AIC	delta AIC	AIC weight
	Intercept	Individual effect			Interactions among variables							
		Viewshed (vs)	Grazing Area (ga)	Density of Crevices (dc)	vs : dc	ga : dc	vs :ga					
22	1.3381	-0.6236	NA	0.8718	NA	-0.5546	NA	7	-90.8404	195.68	0.000	0.146
36	1.4259	-0.5570	NA	0.8259	0.4543	-0.4606	NA	8	-90.0435	196.09	0.406	0.119
21	1.2520	-0.6703	NA	0.5894	0.5641	NA	NA	7	-91.1991	196.40	0.717	0.102
8	1.0777	-0.7859	NA	0.6135	NA	NA	NA	6	-92.3225	196.65	0.964	0.090
38	1.4074	-0.6249	NA	0.8329	NA	-0.4989	0.2294	8	-90.4980	197.00	1.315	0.076
23	1.2199	-0.7643	NA	0.5802	NA	NA	0.3302	7	-91.6754	197.35	1.670	0.063
31	1.3380	-0.6199	0.0100	0.8677	NA	-0.5548	NA	8	-90.8395	197.68	1.998	0.054
45	1.4427	-0.5653	NA	0.8145	0.4036	-0.4469	0.0917	9	-89.9993	198.00	2.318	0.046
41	1.4310	-0.5312	0.0626	0.8018	0.4684	-0.4637	NA	9	-90.0081	198.02	2.335	0.045
37	1.2998	-0.6778	NA	0.5766	0.4689	NA	0.1805	8	-91.0350	198.07	2.389	0.044
30	1.2538	-0.6529	0.0456	0.5723	0.5696	NA	NA	8	-91.1812	198.36	2.682	0.038
17	1.0776	-0.7802	0.0162	0.6075	NA	NA	NA	7	-92.3204	198.64	2.960	0.033
43	1.4358	-0.5656	0.1621	0.7519	NA	-0.4793	0.3358	9	-90.3270	198.65	2.973	0.033
32	1.2639	-0.6852	0.1962	0.4976	NA	NA	0.4518	8	-91.4209	198.84	3.161	0.030
46	1.4691	-0.5175	0.1375	0.7501	0.3819	-0.4389	0.1884	10	-89.8750	199.75	4.069	0.019
42	1.3304	-0.6224	0.1550	0.5121	0.4332	NA	0.2876	9	-90.8781	199.76	4.075	0.019
20	1.4982	-0.6018	0.5526	NA	NA	NA	0.7743	7	-93.8365	201.67	5.992	0.007
34	1.5565	-0.5668	0.5122	NA	0.3986	NA	0.5981	8	-93.3737	202.75	7.067	0.004
18	1.4429	-0.6421	0.4030	NA	0.7077	NA	NA	7	-94.5102	203.02	7.340	0.004
9	1.5035	-0.8306	NA	NA	0.7342	NA	NA	6	-95.6457	203.29	7.611	0.003
35	1.4398	-0.6436	0.5120	NA	NA	0.0934	0.7634	8	-93.7821	203.56	7.883	0.003
11	1.5295	-0.8913	NA	NA	NA	NA	0.5982	6	-96.0337	204.07	8.387	0.002
24	1.3393	-0.8690	NA	NA	0.7447	0.2537	NA	7	-95.1169	204.23	8.553	0.002
25	1.5959	-0.8235	NA	NA	0.5480	NA	0.3698	7	-95.1689	204.34	8.657	0.002
44	1.4972	-0.6106	0.4623	NA	0.4105	0.1043	0.5892	9	-93.3010	204.60	8.921	0.002
7	1.2347	-0.7547	0.4366	NA	NA	NA	NA	6	-96.3531	204.71	9.025	0.002
26	1.3267	-0.9547	NA	NA	NA	0.2983	0.5916	7	-95.4010	204.80	9.121	0.002
33	1.3782	-0.6867	0.3468	NA	0.7097	0.1108	NA	8	-94.4333	204.87	9.186	0.001
39	1.4262	-0.8665	NA	NA	0.5599	0.2802	0.3959	8	-94.5304	205.06	9.380	0.001
1	1.2937	-0.9385	NA	NA	NA	NA	NA	5	-97.6591	205.32	9.637	0.001
3	1.1897	NA	NA	0.6446	NA	NA	NA	5	-97.9055	205.81	10.130	0.001
10	1.0808	-1.0096	NA	NA	NA	0.3165	NA	6	-97.0083	206.02	10.336	0.001
13	1.3878	NA	NA	0.6266	NA	NA	0.4100	6	-97.0774	206.15	10.474	0.001
28	1.8200	NA	0.8864	NA	NA	-0.2031	0.9547	7	-96.0977	206.20	10.515	0.001
19	1.1345	-0.8201	0.3689	NA	NA	0.1645	NA	7	-96.2127	206.43	10.745	0.001
40	1.8548	NA	0.8106	NA	0.5439	-0.1289	0.7156	8	-95.3281	206.66	10.975	0.001
4	1.4473	NA	NA	NA	0.7796	NA	NA	5	-98.8190	207.64	11.957	0.000
27	1.7466	NA	0.7432	NA	0.9351	-0.1573	NA	7	-96.9081	207.82	12.135	0.000
6	1.3896	NA	NA	NA	NA	NA	0.5369	5	-99.4153	208.83	13.150	0.000
15	1.5454	NA	NA	NA	0.6282	NA	0.3372	6	-98.4189	208.84	13.157	0.000
2	1.2007	NA	0.4654	NA	NA	NA	NA	5	-99.6014	209.20	13.522	0.000
14	1.4492	NA	NA	NA	0.7750	-0.0086	NA	6	-98.8185	209.64	13.956	0.000
12	1.3096	NA	0.5183	NA	NA	-0.2257	NA	6	-99.2819	210.56	14.883	0.000
16	1.4191	NA	NA	NA	NA	-0.0788	0.5233	6	-99.3679	210.74	15.055	0.000
29	1.5426	NA	NA	NA	0.6386	0.0194	0.3403	7	-98.4161	210.83	15.151	0.000
5	1.2005	NA	NA	NA	NA	-0.1289	NA	5	-100.502	211.00	15.323	0.000

Supplementary Table S2. Model selection table for *Stegastes partitus*. The table show the estimated coefficients, degrees of freedom, LogLik, and Akaike Information criterion for each model where the combination of explanatory variables was evaluated during model simplification. The models are ordered by AIC values, where the selected model is the one in the first row, with AIC values closest to zero, delta AIC zero and maximum AIC weight, when compared against all other model combinations. "NA" values in the Model coefficients columns indicate that that specific variable or interaction of variables was not included in the model as part of the iterations for model simplification.

**Model ID**	**Model coefficients for selected variables**							**Degrees of Freedom (df)**	**logLik**	**AIC**	**delta AIC**	**AIC weight**
	Intercept	Individual effect			Interactions between variables							
		Viewshed (vs)	Grazing Area (ga)	Density of Crevices (dc)	vs : dc	ga : dc	vs :ga					
2	2.6549	NA	-0.2642	NA	NA	NA	NA	6	-145.658	303.32	0.00	0.125
13	2.7277	NA	-0.2652	NA	0.1525	NA	NA	7	-145.243	304.49	1.17	0.070
14	2.7126	NA	-0.2375	NA	NA	-0.1031	NA	7	-145.38	304.76	1.44	0.061
12	2.6520	NA	-0.2393	-0.0540	NA	NA	NA	7	-145.553	305.11	1.79	0.051
15	2.6837	NA	-0.2505	NA	NA	NA	0.0528	7	-145.555	305.11	1.79	0.051
7	2.6541	0.0171	-0.2574	NA	NA	NA	NA	7	-145.648	305.30	1.98	0.047
21	2.7690	0.1387	-0.2298	NA	0.2641	NA	NA	8	-144.867	305.73	2.42	0.037
28	2.7114	NA	-0.2476	-0.0684	0.1422	NA	NA	8	-145.083	306.17	2.85	0.030
31	2.7279	NA	-0.2602	NA	0.1019	-0.0528	NA	8	-145.201	306.40	3.09	0.027
32	2.7194	NA	-0.2777	NA	0.1759	NA	-0.0312	8	-145.224	306.45	3.13	0.026
22	2.7144	0.0359	-0.2217	NA	NA	-0.1095	NA	8	-145.342	306.68	3.37	0.023
30	2.6921	NA	-0.2095	-0.0775	NA	NA	0.0759	8	-145.358	306.72	3.40	0.023
33	2.7196	NA	-0.2341	NA	NA	-0.0926	0.0232	8	-145.363	306.73	3.41	0.023
29	2.7054	NA	-0.2302	-0.0220	NA	-0.0925	NA	8	-145.366	306.73	3.42	0.023
5	2.8138	NA	NA	NA	NA	-0.1929	NA	6	-147.428	306.86	3.54	0.021
23	2.6981	0.0650	-0.2161	NA	NA	NA	0.0850	8	-145.457	306.91	3.60	0.021
3	2.6765	NA	NA	-0.1616	NA	NA	NA	6	-147.524	307.05	3.73	0.019
20	2.6519	0.0042	-0.2380	-0.0531	NA	NA	NA	8	-145.552	307.10	3.79	0.019
6	2.8048	NA	NA	NA	NA	NA	0.1563	6	-147.561	307.12	3.81	0.019
9	2.8534	0.2008	NA	NA	0.3477	NA	NA	7	-146.576	307.15	3.84	0.018
11	2.7738	0.2013	NA	NA	NA	NA	0.1925	7	-146.665	307.33	4.01	0.017
4	2.8291	NA	NA	NA	0.2148	NA	NA	6	-147.689	307.38	4.06	0.016
10	2.7849	0.1457	NA	NA	NA	-0.1980	NA	7	-146.863	307.73	4.41	0.014
39	2.7711	0.1398	-0.2280	NA	0.2593	-0.0082	NA	9	-144.865	307.73	4.41	0.014
40	2.7657	0.1376	-0.2343	NA	0.2689	NA	-0.0086	9	-144.865	307.73	4.41	0.014
1	2.6811	0.1372	NA	NA	NA	NA	NA	6	-147.881	307.76	4.45	0.014
47	2.7099	NA	-0.2510	-0.0668	0.1485	NA	-0.0077	9	-145.082	308.16	4.85	0.011
19	2.8439	NA	NA	NA	NA	-0.1405	0.0991	7	-147.149	308.30	4.98	0.010
26	2.8767	0.2108	NA	NA	0.2522	NA	0.1283	8	-146.163	308.33	5.01	0.010
49	2.7215	NA	-0.2722	NA	0.1315	-0.0542	-0.0358	9	-145.175	308.35	5.03	0.010
16	2.7512	NA	NA	-0.1116	NA	-0.1272	NA	7	-147.209	308.42	5.10	0.010
41	2.7325	0.0649	-0.2009	NA	NA	-0.0906	0.0550	9	-145.27	308.54	5.22	0.009
38	2.7047	0.0584	-0.1806	-0.0734	NA	NA	0.1034	9	-145.281	308.56	5.25	0.009
8	2.6697	0.0869	NA	-0.1312	NA	NA	NA	7	-147.283	308.57	5.25	0.009
48	2.7111	NA	-0.2141	-0.0495	NA	-0.0571	0.0494	9	-145.31	308.62	5.30	0.009
27	2.8163	0.1910	NA	NA	NA	-0.1274	0.1420	8	-146.313	308.63	5.31	0.009
17	2.8411	NA	NA	NA	0.0958	-0.1486	NA	7	-147.33	308.66	5.34	0.009
37	2.7106	0.0320	-0.2196	-0.0112	NA	-0.1034	NA	9	-145.339	308.68	5.36	0.009
25	2.8578	0.2016	NA	NA	0.2573	-0.1011	NA	8	-146.374	308.75	5.43	0.008
18	2.8525	NA	NA	NA	0.1372	NA	0.1053	7	-147.423	308.85	5.53	0.008
35	2.7656	NA	NA	-0.1642	NA	-0.0259	0.1393	8	-146.716	309.43	6.12	0.006
24	2.7612	0.1221	NA	-0.0566	NA	-0.1626	NA	8	-146.779	309.56	6.24	0.006
52	2.7675	0.1388	-0.2331	NA	0.2642	-0.0097	-0.0104	10	-144.863	309.73	6.41	0.005
43	2.8512	0.2073	NA	-0.0934	0.2339	NA	0.1309	9	-145.89	309.78	6.46	0.005
44	2.7767	0.1576	NA	-0.1150	NA	-0.0397	0.1780	9	-145.997	309.99	6.68	0.004
45	2.8869	0.2095	NA	NA	0.2260	-0.0573	0.1139	9	-146.087	310.17	6.86	0.004

Supplementary Table S3. Model selection table for *Stegastes planifrons*. The table show the estimated coefficients, degrees of freedom, LogLik, and Akaike Information criterion for each model where the combination of explanatory variables was evaluated during model simplification. The models are ordered by AIC values, where the selected model is the one in the first row, with AIC values closest to zero, delta AIC zero and maximum AIC weight, when compared against all other model combinations. "NA" values in the Model coefficients columns indicate that that specific variable or interaction of variables was not included in the model as part of the iterations for model simplification.

**Model ID**	**Model coefficients for selected variables**							**Degrees of Freedom (df)**	**logLik**	**AIC**	**delta AIC**	**AIC weight**
	Intercept	Individual effect			Interactions among variables							
		Viewshed (vs)	Grazing Area (ga)	Density of Crevices (dc)	vs : dc	ga : dc	vs :ga					
53	-0.3541	-2.0347	0.9965	1.1804	NA	-0.7378	1.2514	9	-52.3282	122.66	0.00	0.1498
8	-1.6334	-0.9616	NA	0.8101	NA	NA	NA	6	-55.5689	123.14	0.48	0.1177
25	-1.2877	-1.3321	NA	0.9815	NA	-0.5272	NA	7	-54.6963	123.39	0.74	0.1036
24	-1.4286	-1.0687	NA	1.0838	0.3807	NA	NA	7	-55.0639	124.13	1.47	0.0718
26	-1.5386	-1.0761	NA	0.8559	NA	NA	0.2026	7	-55.0674	124.13	1.48	0.0715
40	-0.8392	-1.4894	0.6326	0.9443	NA	NA	0.9675	8	-54.317	124.63	1.98	0.0557
43	-1.3299	-1.2764	NA	0.8968	-0.2149	-0.5191	NA	8	-54.4078	124.82	2.16	0.0509
21	-1.6623	-0.9835	-0.0866	0.8351	NA	NA	NA	7	-55.4245	124.85	2.19	0.0500
56	-1.0071	-1.7181	0.9104	0.9169	-0.3620	-0.6771	1.2194	10	-52.5442	125.09	2.43	0.0444
45	-1.3894	-1.1696	NA	0.9786	NA	-0.2620	0.1445	8	-54.6142	125.23	2.57	0.0414
39	-1.2698	-1.3345	0.0313	0.9851	NA	-0.5394	NA	8	-54.6803	125.36	2.70	0.0387
44	-1.4148	-1.1351	NA	1.0476	0.2832	NA	0.1527	8	-54.803	125.61	2.95	0.0343
52	-0.9693	-1.5006	0.5862	1.1169	0.2929	NA	0.8576	9	-54.0088	126.02	3.36	0.0279
38	-1.4781	-1.0671	-0.0466	1.0671	0.3453	NA	NA	8	-55.0254	126.05	3.39	0.0274
54	-1.3337	-1.3031	NA	0.8805	-0.2391	-0.4985	0.1310	9	-54.242	126.48	3.83	0.0221
51	-1.3146	-1.2787	0.0261	0.9005	-0.2135	-0.5296	NA	9	-54.3967	126.79	4.14	0.0189
3	-2.2334	NA	NA	0.4748	NA	NA	NA	5	-58.6976	127.40	4.74	0.0140
15	-2.0969	NA	NA	0.6109	0.3104	NA	NA	6	-58.3031	128.61	5.95	0.0076
17	-2.1736	NA	NA	0.4707	NA	NA	0.0951	6	-58.5626	129.13	6.47	0.0059
11	-2.2478	NA	-0.0440	0.4823	NA	NA	NA	6	-58.6502	129.30	6.64	0.0054
16	-2.2510	NA	NA	0.4663	NA	0.0157	NA	6	-58.6955	129.39	6.73	0.0052
34	-2.1794	NA	NA	0.5824	0.3623	0.1026	NA	7	-58.2261	130.45	7.80	0.0030
35	-2.0831	NA	NA	0.5947	0.2801	NA	0.0458	7	-58.275	130.55	7.89	0.0029
28	-2.1085	NA	-0.0214	0.6091	0.2996	NA	NA	7	-58.2921	130.58	7.93	0.0028
30	-2.0570	NA	0.1249	0.4438	NA	NA	0.2406	7	-58.4915	130.98	8.33	0.0023
36	-2.2391	NA	NA	0.4341	NA	0.0698	0.1139	7	-58.5244	131.05	8.39	0.0023
29	-2.3239	NA	-0.0605	0.4531	NA	0.0616	NA	7	-58.6235	131.25	8.59	0.0020
5	-2.6202	NA	NA	NA	NA	0.3087	NA	5	-60.7495	131.50	8.84	0.0018
50	-2.1779	NA	NA	0.5521	0.3257	0.1253	0.0695	8	-58.164	132.33	9.67	0.0012
46	-2.2388	NA	-0.0513	0.5696	0.3518	0.1372	NA	8	-58.1719	132.34	9.69	0.0012
4	-2.3728	NA	NA	NA	-0.3243	NA	NA	5	-61.1761	132.35	9.70	0.0012
41	-1.6125	-0.8094	0.5191	NA	-0.5710	NA	0.8370	8	-58.2241	132.45	9.79	0.0011
47	-2.0334	NA	0.0582	0.5747	0.2628	NA	0.1168	8	-58.2613	132.52	9.87	0.0011
1	-1.8595	-0.4123	NA	NA	NA	NA	NA	5	-61.2751	132.55	9.89	0.0011
9	-2.3407	-0.2948	NA	NA	NA	0.2988	NA	6	-60.3214	132.64	9.99	0.0010
20	-2.5684	NA	NA	NA	NA	0.3479	0.1534	6	-60.381	132.76	10.11	0.0010
48	-2.1135	NA	0.1083	0.4224	NA	0.0468	0.2337	8	-58.4757	132.95	10.30	0.0009
13	-2.6987	NA	-0.0735	NA	NA	0.3487	NA	6	-60.6227	133.25	10.59	0.0008
18	-2.6242	NA	NA	NA	-0.0752	0.2732	NA	6	-60.7244	133.45	10.79	0.0007
6	-2.1565	NA	NA	NA	NA	NA	0.0839	5	-61.7563	133.51	10.86	0.0007
2	-2.1888	NA	-0.0027	NA	NA	NA	NA	5	-61.8639	133.73	11.07	0.0006
27	-2.2939	-0.3040	NA	NA	NA	0.3397	0.1662	7	-59.9154	133.83	11.17	0.0006
19	-2.3263	NA	NA	NA	-0.3575	NA	0.1297	6	-60.9476	133.90	11.24	0.0005
10	-1.6035	-0.7106	NA	NA	NA	NA	0.1224	6	-60.9636	133.93	11.27	0.0005
23	-1.5898	-0.5000	0.4680	NA	NA	NA	0.6620	7	-60.0986	134.20	11.54	0.0005
12	-2.3968	NA	-0.0624	NA	-0.3566	NA	NA	6	-61.099	134.20	11.54	0.0005
